# RPA homologs and ssDNA processing during meiotic recombination

**DOI:** 10.1007/s00412-015-0552-7

**Published:** 2015-10-31

**Authors:** Jonathan Ribeiro, Emilie Abby, Gabriel Livera, Emmanuelle Martini

**Affiliations:** Laboratory of Development of the Gonads, Unit of Stem Cells and Radiation, University of Paris Diderot, Sorbonne Paris Cité, UMR 967, F-92265 Fontenay-aux-Roses, France; CEA, DSV, iRCM, SCSR, LDG, F-92265 Fontenay-aux-Roses, France; INSERM, Unité 967, F-92265 Fontenay-aux-Roses, France; Université Paris-Saclay, UMR-967, F-92265 Fontenay-aux-Roses, France

**Keywords:** Meiosis, Recombination, ssDNA, RPA, MEIOB, SPATA22

## Abstract

Meiotic homologous recombination is a specialized process that involves homologous chromosome pairing and strand exchange to guarantee proper chromosome segregation and genetic diversity. The formation and repair of DNA double-strand breaks (DSBs) during meiotic recombination differs from those during mitotic recombination in that the homologous chromosome rather than the sister chromatid is the preferred repair template. The processing of single-stranded DNA (ssDNA) formed on intermediate recombination structures is central to driving the specific outcomes of DSB repair during meiosis. Replication protein A (RPA) is the main ssDNA-binding protein complex involved in DNA metabolism. However, the existence of RPA orthologs in plants and the recent discovery of meiosis specific with OB domains (MEIOB), a widely conserved meiosis-specific RPA1 paralog, strongly suggest that multiple RPA complexes evolved and specialized to subdivide their roles during DNA metabolism. Here we review ssDNA formation and maturation during mitotic and meiotic recombination underlying the meiotic specific features. We describe and discuss the existence and properties of MEIOB and multiple RPA subunits in plants and highlight how they can provide meiosis-specific fates to ssDNA processing during homologous recombination. Understanding the functions of these RPA homologs and how they interact with the canonical RPA subunits is of major interest in the fields of meiosis and DNA repair.

**Electronic supplementary material:**

The online version of this article (doi:10.1007/s00412-015-0552-7) contains supplementary material, which is available to authorized users.

Meiosis is the central process of sexual reproduction. During this specialized cell division program, the genome of diploid germ cells is halved to produce haploid gametes. This process requires the pairing and formation of a physical link (chiasma) between homologous chromosomes (homologs). These events are established during meiotic prophase I, which is subdivided into four stages (for review see Page and Hawley 2003). Prophase begins with the formation of programmed DNA double-strand breaks (DSBs) during the leptotene stage. During the zygotene and pachytene stages, these DSBs are progressively repaired by homologous recombination (HR), a process that promotes and completes homolog pairing (Weiner and Kleckner 1994; Kauppi et al. 2013). Chiasmata are the product of crossovers (COs) formed during the pachytene stage. Only a subset of DSBs are repaired with a reciprocal exchange of chromosome arms to form a CO, whereas the remaining DSBs are repaired without reciprocal exchange to form noncrossovers (NCOs). COs are tightly regulated as shown by their controlled number and non-random distribution along chromosomes (Anderson et al. 1999; Martini et al. 2006; Cole et al. 2012). It is now evident that numerous factors are necessary to control the proper progression of meiotic HR and drive the maturation of intermediates to specific outcomes (for review seeYouds and Boulton 2011). First, meiotic DSBs are programmed and formed through the action of several factors including the conserved topoisomerase-like transesterase SPO11, which bears the catalytic activity. DSB initiation was recently shown to be under feedback control by Tel1/ATM kinase (Carballo et al. 2013; Lange et al. 2011; Garcia et al. 2015). During a later step, the conversion of pre-crossover intermediates into COs has been demonstrated to depend on the coordination of a number of proteins (De Muyt et al. 2014; Holloway et al. 2014; Qiao et al. 2014).

Single-stranded DNA (ssDNA) is generated at multiple steps during HR (see Fig. 1) and requires specific factors for its formation, signal transduction, protection, and disappearance. Among these factors, Replication Protein A (RPA) is the principal ssDNA-binding complex and is essential for mitotic growth and meiotic progression (Soustelle et al. 2002). However, recent studies have highlighted the involvement of additional RPA homologs specifically during meiosis, notably meiosis specific with OB domains (MEIOB) a new meiosis-specific paralog of the largest subunit of RPA (Souquet et al. 2013; Luo et al. 2013). In this review we discuss the potential specific roles of RPA and its homologs to better understand the regulation of ssDNA-containing meiotic recombination intermediates.

**Fig. 1:**
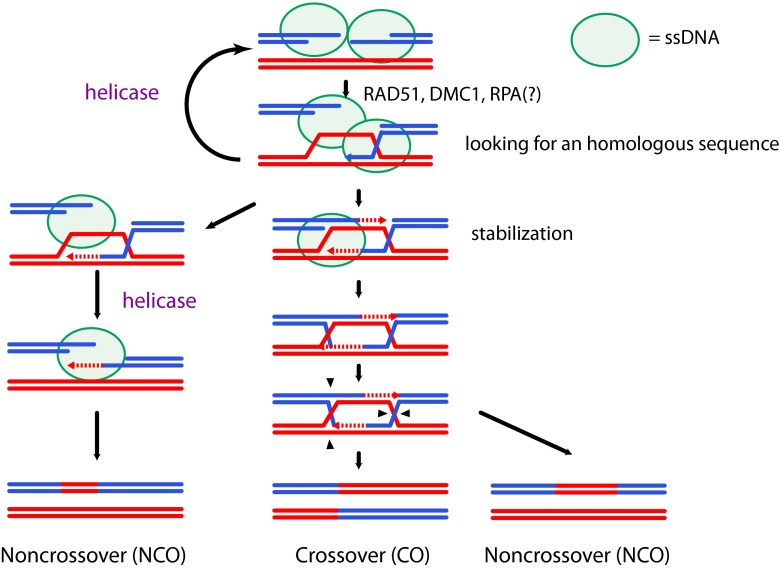
Meiotic recombination and ssDNA. Meiotic double strand breaks are repaired as crossovers (CO) or non-crossovers (NCOs) through different intermediates. After DSBs formation, resection is initiated to form 3′-ssDNA tails. The 3′-tail is then coated by the recombinases to invade the homologous sequence on the homolog. A ssDNA-binding protein such as RPA must help to stabilize and protect ssDNA before the formation of the presynaptic filament. Proper strand invasion is stabilized to initiate DNA synthesis and then either further stabilized or destabilized to be repaired by synthesis-dependent strand annealing (SDSA). The stabilized intermediates (double Holliday junctions) can either form a NCO outcome or be resolved by nuclease activity to form a CO or a NCO outcome. *Green circles* show ssDNA

## Roles of ssDNA during homologous recombination

### During the mitotic cell cycle

ssDNA formation is a common feature of DNA metabolism. It is generated during DNA replication, repair, and recombination (Mehta and Haber 2014). During HR, ssDNA is formed on different intermediates and bound by dedicated proteins that ensure its protection and signaling and the subsequent steps of DNA processing. HR begins with the production of 3′-ssDNA tails to invade duplex DNA during the homology search. Subsequently, D-loop formation and migration generate additional ssDNA. Shortly after formation, DSBs are recognized by the MRN complex (MRE11, RAD50, and NBS1) to initiate 5′- > 3′- resection (Ivanov et al. 1994; Tsubouchi and Ogawa 1998). To protect ssDNA from degradation and remove secondary structures that would prevent the loading of recombinase proteins, the RPA complex binds the resected DNA (Alani et al. 1992; Sugiyama et al. 1997; Wang and Haber 2004). RPA is subsequently replaced by the RAD51 recombinase to form a presynaptic nucleofilament in a BRCA2- and Rad52-dependent manner in mammals and yeast, respectively, with the help of RAD51 paralogs (Sung 1997; Gasior et al. 2001; Jensen et al. 2010; Suwaki et al. 2011; Gaines et al. 2015). In eukaryotic cells, it is assumed that RPA loading precedes RAD51 loading. However, RAD51 can be loaded on ssDNA in the absence of RPA if the ssDNA is free of secondary structures (Heyer and Kolodner 1989; Sung 1994; Sugiyama et al. 1997; Gaines et al. 2015). The large subunit of RPA interacts with RAD51 through its N-terminal region and co-localizes to sites of DNA damage; however, it remains unclear whether RPA and RAD51 coexist in the nucleofilament (Golub et al. 1998; Tarsounas et al. 2003; Haber 2014).

Owing to its DNA strand-pairing activity, the nucleofilament invades a homologous DNA duplex to initiate DNA synthesis from the invading 3′-OH end using the donor sequence as the template. Genetic evidences have revealed that the mismatch repair machinery (MMR) inhibits recombination between moderately divergent regions (Evans and Alani 2000). Studies with mismatches containing substrates incubated in human cell extracts have shown that RPA is recruited at mismatch sites prior to MMR-specific members (Guo et al. 2006). Moreover, the in vitro reconstitution of MMR reaction with purified hMMR proteins has provided evidence that RPA forms part of the complex that initiates mismatch-provoked excision, suggesting that RPA plays a role in the early steps of the MMR. It remains unclear whether RPA collaborates with the MMR machinery in mismatch tolerance during the search for homologous sequences that occurs during HR. Furthermore, whether RPA ensures this role on the nucleofilament or after loading onto the D-loop formed during strand exchange is an open question. Evidence for the presence of RPA on the D-loop has been provided by several observations (Wang and Haber 2004). Biochemical assays showed that RPA strongly enhances strand exchange by stabilizing RAD51-dependent pairing after strand invasion and stimulates DNA synthesis through its interaction with DNA polymerases alpha and delta after invasion (Eggler et al. 2002; Sneeden et al. 2013). RPA-dependent strand invasion stabilization is achieved by preventing reversal of the strand exchange reaction. Chromatin immunoprecipitation of RPA performed in *Saccharomyces cerevisiae* revealed that RPA is retrieved on both the donor and the recipient sequences with time differences confirming the presence of RPA first on the broken strand and then on the D-loop in vivo (Wang and Haber 2004). Moreover, in vitro studies have indicated that RPA favors strand annealing directed by yeast or human RAD52 (Sugiyama et al. 1998; Jensen et al. 2010). Finally, the importance of RPA during second-end capture has been demonstrated by biochemical assays performed with the *S. cerevisiae rfa1-t11* mutant. This mutant supports the RAD52-dependent loading of RAD51 and strand invasion but exhibited failure in second-end capture (Sugiyama et al. 2006).

### During prophase I of meiosis

Even though meiotic and mitotic HR share common features and factors, several facets of HR differ. DSBs are programmed during meiosis and involve the presence of specific factors at DSB sites, including before DSB formation. Our knowledge regarding the proteins required for meiotic DSB formation comes mostly from studies performed in *S. cerevisiae*, which has at least nine proteins that are essential to this process, whereas only three have so far been identified in mice. In *S. cerevisiae*, Mer2 is loaded onto chromatin to coordinate DNA replication and DSB formation in a phosphorylation-dependent manner (Henderson et al. 2006; Murakami and Keeney 2014). The remaining proteins are subsequently loaded to induce DSB formation through the catalytic activity of Spo11. Interestingly, the MRX complex (Mre11, Rad50, and Xrs2) which is involved in ssDNA formation to produce an ssDNA 3′ end during mitotic HR, is essential for DSB formation in *S. cerevisiae* and *Caenorhabditis elegans* (Borde 2007). This observation indicates that a complex loaded on broken ends during mitotic HR to initiate ssDNA processing is present before DSB formation during meiotic HR. Consequently, the chromatin context in which ssDNA processing occurs differs between both types of HR. Interestingly, although RPA foci are detected on unsynapsed chromosomes suggesting the presence of RPA on ssDNA before strand invasion, the precocious formation of numerous and bright RAD51 foci at the time the first RPA foci are detected may bring into question whether RPA binding on ssDNA precedes the loading of recombinases during meiotic HR in mammals as observed during mitotic HR (Plug et al. 1998; Moens et al. 2002; Oliver-Bonet et al. 2007 and personal observation E.A., EM). Of note, RPA foci have been observed on chromosome spreads performed in a *rad50S* mutant of *S. cerevisiae*. This mutant is unable to process DNA after DSB formation and is not expected to produce ssDNA suggesting that normal resection is not required to recruit RPA to DSB sites (Gasior et al. 1998). This finding could be explained through the direct interaction described between MRE11 and RPA or by nonspecific DNA degradation (Gasior et al. 1998).

Unlike mitotic recombination, meiotic recombination exhibits preference to use as the repair template a DNA sequence present on the homologous chromosome rather than a DNA sequence on the sister chromatid and favors the formation of COs (Schwacha and Kleckner 1994; Cole et al. 2014). In addition to RAD51, most organisms possess the meiosis-specific DMC1 recombinase, which is essential together with RAD51 for proper strand invasion (Bishop et al. 1992; Yoshida et al. 1998). Studies performed in *S. cerevisiae* and *Arabidopsis thaliana* suggest that during meiotic HR, RAD51 would preferentially play an accessory role to allow DMC1 recombinase activity (Cloud et al. 2012; Da Ines et al. 2013). These observations suggest that RAD51 and DMC1 can load together on both broken ends after DSB formation. However, the distances observed between RAD51 and DMC1 foci by immunolocalization in *A. thaliana* support the hypothesis that distinct filaments would be formed on each end (Kurzbauer et al. 2012). Although a strong effort has been made to decipher the formation, maintenance, and dynamics of the RAD51/DMC1 nucleofilament, further investigations remain necessary (Brown and Bishop 2014). Studies performed in plants and mammals indicate that BRCA2 interacts with DMC1 and RAD51, suggesting that BRCA2 promotes the formation of the meiotic nucleofilament containing RAD51 and DMC1 (Siaud et al. 2004; Thorslund and West 2007; Seeliger et al. 2012). Genetic evidence has confirmed the importance of BRCA2 to mediate the proper formation of the meiotic presynaptic nucleofilament (Sharan et al. 2004). In addition to stimulating nucleofilament formation, yeast RAD52 (yRad52) is required for the post-invasion steps of meiotic HR (Lao et al. 2008). Indeed, the annealing of the second broken end during synthesis-dependent strand annealing (SDSA) and second-end capture to complete the formation of double Holliday junctions (dHJs) are dependent on yRad52 in *S. cerevisiae* and BRCA-2 in *C. elegans* (Sugiyama et al. 2006; Petalcorin et al. 2006). However, in vitro assays have demonstrated that hBRCA2 does not anneal RPA-coated ssDNA, whereas hRAD52 does, suggesting that two proteins are needed in mammals compared with one in yeast and *C. elegans* (Jensen et al. 2010; Petalcorin et al. 2006). Nonetheless, in contrast to what has been described in yeast, knockouts of the *Rad52* gene in mammals show few phenotypes with no obvious defect in response to DNA damaging agents and no meiotic defects (Rijkers et al. 1998).

The presence of recombinases on the presynaptic filament is essential for the displacement and invasion of the homologous sequence. However, the nature, organization, and dynamics of the nucleofilament formed on the second broken end remain to be determined in vivo. Given the specific outcomes of this end (i.e., strand annealing or second-end capture) it is unclear whether the recombinases and/or RPA are loaded on that end. Immunostaining of RPA performed on chromosome spreads in numerous species (mammals and plants) have shown that RPA foci are detected up to the pachytene stage, specifically until the formation of MLH1 CO-associated foci (Plug et al. 1998). This finding suggests that RPA is present on joint molecules after their stabilization with the MutS homologs, MSH4-MSH5. Interestingly, the phosphoregulation of RPA appears to play a role in the control of CO formation. In *S. cerevisiae,* the phosphorylation of the RPA2 subunit is controlled by the yeast ATR ortholog, Mec1. A phosphomimetic form of RPA2 induces changes in genetic distances and CO interference, suggesting a role for RPA2 phosphorylation in CO regulation (Bartrand et al. 2006).

For decades, RPA has been considered a strong unalterable complex that is well conserved among species. However, the identification in plants of variants of the three subunits of RPA that can form different complexes with specific activities strongly underlines the potential regulatory role that such complexes can play (Shultz et al. 2007). The existence of alternative RPA complexes in mammalian cells has been suggested by the discovery of RPA4, which bears 50 % identity with RPA2, in a cDNA library derived from HeLa cells (Keshav et al. 1995). More recently, it has been proposed that RPA4 is more likely to be involved in the maintenance of genomic integrity than in its replication (Haring et al. 2010). Moreover, recent studies identified a new meiosis-specific gene in metazoans, MEIOB, a paralog of RPA1 that interacts with RPA2 (Souquet et al. 2013; Luo et al. 2013). MEIOB is essential for proper meiotic recombination in mice and interacts with ssDNA. In-depth characterization of these multiple complexes should allow a better understanding of the mechanisms regulating meiotic recombination and the maintenance of genome integrity.

## RPA the major ssDNA-binding protein

### Canonical RPA complex

The ssDNA-binding replication protein A (RPA) is an evolutionarily conserved heterotrimeric complex involved in DNA replication, repair, and recombination which is essential for cell survival (for a review see Wold 1997). RPA was originally identified through its essential role for SV40 DNA replication in vitro (Wold and Kelly 1988).

**Fig. 2:**
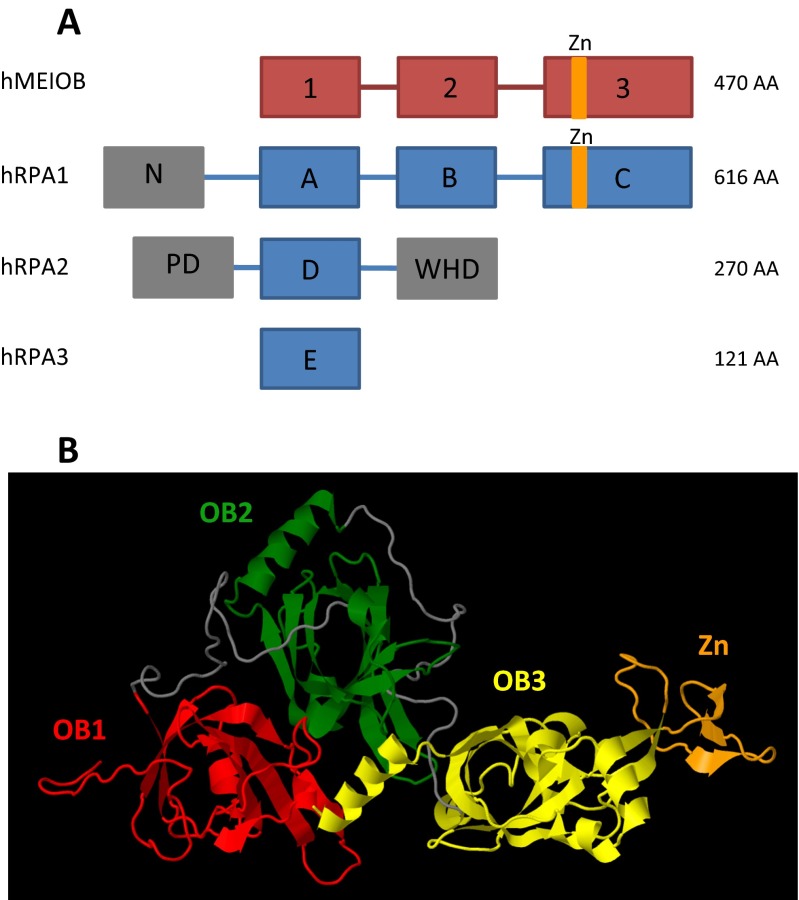
Domain structures of RPA subunits and MEIOB. **a** Schematic representation of MEIOB and RPA subunits protein domains. The folded domains and unfolded linkers are presented as *boxes* and *lines* respectively. The *red* and *blue boxes* represent MEIOB and RPA subunit OB- folds, respectively. The *orange* domains illustrate zinc ion-binding domains. The *grey boxes* represent domains that are not involved in ssDNA-binding activity, such as the N-terminal domain of RPA1 and the phosphorylation domain (PD) and the winged-helix domain (WHD) of RPA2. **b** Stereo ribbon presentation of predicted MEIOB structure model obtained from the RaptorX server (Kallberg et al. 2012) and visualized with Jmol (www.jmol.org). OB-folds 1, 2, and 3 and zinc ion-binding domain are represented in *red*, *green*, *yellow*, and *orange*, respectively. Unfolded linkers are represented in *grey*

The RPA complex is composed of three subunits: RPA1, RPA2, and RPA3. X-ray diffraction studies of the crystallized human complex revealed that the three subunits form together a trimer that is stabilized through the trimerization core produced by the interaction of helices from each subunit (Bochkareva et al. 2002). Among the three subunits, RPA1 possesses the highest affinity for ssDNA through its three oligonucleotide/oligosaccharide-binding folds (OB-fold), denoted DBD-A, DBD-B and DBD-C (Wold 1997). RPA2 and RPA3 also possess OB-fold domains, named DBD-D and DBD-E, which are capable of interacting with ssDNA (Philipova et al. 1996; Salas et al. 2009) (see Fig. 2). RPA binds to ssDNA in two conformational states that differ in the length and affinity of the bound DNA. A study of the structure of a crystallized RPA-ssDNA complex from the fungus *Ustilago maydis* demonstrated that the two conformational states of RPA-ssDNA binding provides opposing affinities for DNA and proteins (Fan and Pavletich 2012). The DBD-C of RPA1 is longer than the two other OB-folds and possesses an insertion of approximately 30 amino acids with a conserved zinc ion-binding domain of type C-4X-C-13X-C-2X-C (Heyer et al. 1990; Erdile et al. 1991). This domain is dispensable for RPA1 ssDNA-binding activity but influences the overall ssDNA-binding affinity of the RPA complex (Kim et al. 1996; Dong et al. 1999; Walther et al. 1999). However, the functional role of this domain remains poorly understood. Kim et al. and Walther et al. reported that mutating this domain strongly impaired SV40-dependent DNA replication, whereas Dong et al. observed only a slight delay.

### Existence and properties of multiple RPA complexes

Genome duplications occurred periodically during eukaryotic evolution such that some organisms possess several homologs of RPA subunits that may have meiosis-specific functions. Such multiple RPAs have been described in the plants *A. thaliana* and *Oryza sativa* and in the protists *Toxoplasma gondii*, *Plasmodium falciparum,* and *Cryptosporidium parvum* (Rider et al. 2005; Gopalakrishnan and Kumar 2013).

The flowering plant *A. thaliana* contains five homologs of RPA1, two homologs of RPA2, and two homologs of RPA3. The rice *O. sativa* possesses three homologs of RPA1 and RPA2 and only one homolog of RPA3. Interestingly, despite the multiple possible combinations, only three complexes have been identified in rice and four in *A. thaliana*, respectively (Ishibashi et al. 2006; Eschbach and Kobbe 2014). These observations suggest the existence of specific interactions between subunits and/or specialized expression that restricts the possibilities of interactions.

Genetic studies performed with mutants of AtRPA1 homologs led to the classification of these homologs into two groups. AtRPA1a, AtRPA1c, and AtRPA1e are hypothesized to be involved in the response to DNA-damaging agents and consequently in DNA repair, whereas AtRPA1b and AtRPA1d are proposed to be necessary for normal DNA replication during developmental growth (Aklilu et al. 2014). No meiotic defect could be observed in *Atrpa1b,c,d,e* mutants (Aklilu et al. 2014). A careful genetic study of *AtRPA1a* during meiosis revealed its role in meiotic HR. Although the absence of fragmented DNA suggests that DSBs are repaired in this mutant, immunostaining performed on meiotic chromosome spreads showed a reduction in the MLH1 foci number, strongly suggesting a diminution of interfering COs (class I). Metaphase I analysis confirmed a reduction in the chiasma number. The observed phenotype suggested that meiosis progressed normally until class I CO formation. Therefore, the authors proposed that AtRPA1a could be involved in the second-end capture of the broken end at the recombination intermediates designated to form class I COs controlled by MLH1/MLH3 (Osman et al. 2009). Furthermore, mutation of both *AtRPA1c* and *AtRPA1a* was found to induce complete sterility and marked chromosome fragmentation during meiosis. The findings that the *Atrpa1a/Atrpa1c* double mutant is sterile and that the *Atrpa1c* mutant does not show fertility problems suggest that AtRPA1c could play an early role during meiotic recombination that could be overcome by AtRPA1a in the absence of AtRPA1c.

Less genetic data are available regarding the functions of RPA1 homologs in rice. To date, only the function of OsRPA1a has been studied. The *osrpa1a* mutant showed normal vegetative growth but an increased sensitivity to DNA damage induced by genotoxic agents, including UV irradiation. *osrpa1a* mutants are sterile and show chromosomal fragmentation despite normal chromosome pairing and synapsis during meiosis (Chang et al. 2009). These data suggest that OsRPA1a is dispensable for replication but required for somatic and meiotic DNA repair.

Not much is known about the biochemical properties of the RPA complexes in rice, however, the DNA binding properties of AtRPA complexes were recently published (Eschbach and Kobbe 2014). The authors concluded that complexes containing AtRPA1a show higher affinity for unmodified ssDNA than ssDNA with modifications, such as abasic sites, and stronger dsDNA-destabilizing activity than AtRPA1b-containing complexes. These results were unexpected in light of genetic data suggesting that AtRPA1b is specifically involved in DNA replication because the *Atrpa1a* mutant did not show growth defects in the absence of DNA-damaging agents. Such results may be explained by the absence of RPA-interacting partners or post-translational modifications in the in vitro assay. In addition, the study revealed that the identity of the RPA3 homolog may significantly influence the DNA-binding properties of the complexes. This result was unexpected given the weak DNA-binding affinity of RPA3, a subunit that was proposed to mediate protein-protein interaction (Bochkarev et al. 1999).

Collectively, these genetic and biochemical studies suggest that multiple RPA complexes have evolved and become specialized to subdivide their roles during the various stages of DNA metabolism.

## MEIOB: a meiosis-specific RPA1 homolog

### MEIOB and evolution

**Fig. 3:**
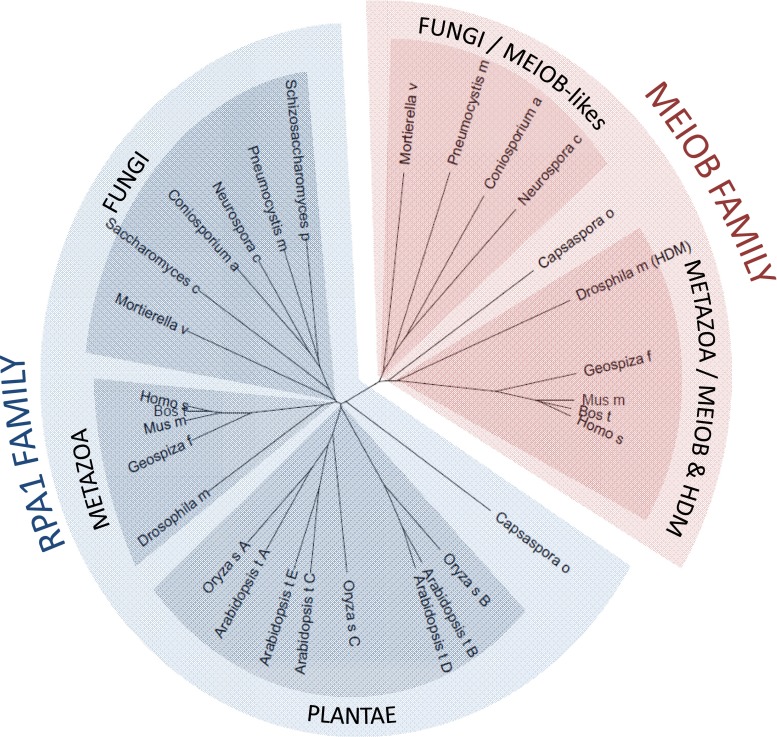
Phylogenetic relationship between RPA and MEIOB homologs. Multiple alignments of full-length MEIOB and RPA1 protein were processed with Clustal Omega (http://www.ebi.ac.uk/Tools/msa/clustalo/). The phylogenetic tree obtained with Clustal Omega was visualized with Archaeopteryx Version 0.9901 beta (Han and Zmasek 2009). MEIOB and RPA1 proteins form distinct families. Represented species: H. sapiens, *Homo sapiens*; B. taurus, *Bos taurus*; M. musculus, *Mus musculus*; G. fortis, *Geospiza fortis*; D. melanogaster, *Drosophilae melanogaster*; C. owczarzaki, *Capsaspora owczarzaki*; N. crassa, *Neurospora crassa*; C. apollinis, *Coniosporium apollinis*; P. murina, *Pneumocystis murina*; M. verticillata, *Mortierella verticillata*; S. pombe, *Schizosaccharomyces pombe*; S. cerevisiae, *Saccharomyces cerevisiae*; O. sativa, *Oryza sativa;* A. thaliana, *Arabidopsis thaliana*. The amino acid sequences and accession numbers are available in supplementary material (Sup. 1)

For several decades, RPA4, which belongs to the RPA2 family, was the only RPA paralog to have been found in mammals. However, recent works identified *Meiob* as a new meiosis-specific paralog of *Rpa1* in metazoans (Souquet et al. 2013; Luo et al. 2013). *Meiob* orthologs have been retrieved in the genomes of almost all metazoans, with the exception of *Nematoda* and a non-meiotic specific ortholog of MEIOB, named Hold’em (HDM), has been described in *Drosophila* (Joyce et al. 2009). Interestingly, an ortholog has also been found in the single-celled organism *Capsaspora owczarzaki* (Souquet et al. 2013). This finding strongly suggests that a duplication event that occurred before the emergence of metazoans and multicellularity enabled *Meiob* to evolve from an ancestral *Rpa1* gene (approximately 600 million years ago). However, we recently identified sequences similar to metazoan *Meiob* in several fungal genomes such as the zygomycotan fungi *Mortierella verticillata*, and in genomes of ascomycetes, such as *Neurospora crassa*, suggesting that the evolution of *Meiob* may be earlier than first thought (22.3 % identity between human MEIOB and MEIOB-like of *M. verticillata,* see Fig. 3, unpublished data). Deciphering the function of these Meiob-like proteins will help us to better understand the role of MEIOB. To date, we have not identified a *Meiob* ortholog in amoebozoan genomes. The fact that MEIOB has been retrieved from metazoans, fungi, and *Capsasporidae* (*C. owczarzaki*) suggests that the appearance of MEIOB occurred during the evolution of the *Opisthokonta* monophyletic group. Surprisingly, putative *Meiob* orthologs have not been identified in the *S. cerevisiae* or *Schizosaccharomyces pombe* genomes. This could be explained by the loss of an ancestral *Meiob* gene during the evolution of these species—a situation that has frequently been observed with other meiotic genes. For example, the meiotic recombinase DMC1 conserved from yeasts to humans is absent from the *C. elegans*, *Drosophila melanogaster*, and *Sordaria macrospora* genomes.

### Structure of MEIOB

MEIOB possesses three OB-folds homologous to those of RPA1 (Souquet et al. 2013; Luo et al. 2013). Similar to RPA1, MEIOB possesses two OB-folds and a long C-terminal OB-fold with a 30-amino acid insertion containing a putative zinc ion-binding domain (see Fig. 2). We identified this motif in the human MEIOB sequence using the Sequence Similarity DataBase of the Kyoto Encyclopedia of Genes and Genomes (KEGG SSDB, http://www.kegg.jp/kegg/ssdb). This C-2X-C-10X-C-2X-C motif resembles that of a double zinc ribbon (DZR, unpublished data) and differs from that present in RPA1 only in the number of residues between the cysteines. We identified this motif in all metazoan MEIOB as well as in the MEIOB-like protein of the *M. verticillata*. The existence of this motif reinforces the hypothesis of a common origin between MEIOB and RPA1 even though MEIOBs and MEIOB-likes form a monophyletic group distinct from RPA1. The role of the putative zinc ion-binding domain of MEIOB should be the subject of future work to specify MEIOB functions during meiotic HR.

Compared with the very similar OB-fold domains, RPA1 and MEIOB significantly differ in the N-terminal parts of the proteins. The well-conserved N-terminal domain of RPA1 is absent in MEIOB. This domain ensures specific interactions between RPA1 and numerous partners such as DNA polymerase alpha and checkpoint proteins including ATRIP, RAD9 (53BP1), the MRE11/RAD50/NBS1 complex, and the tumor suppressor p53 (Kim et al. 1996; Bochkareva et al. 2002; Xu et al. 2003). This absence of such an N-terminal domain suggests that MEIOB developed its own specific interactions with a limited set of partners. This difference may support the existence of a distinct role for MEIOB and partially explain why RPA1 cannot compensate for the absence of MEIOB during meiosis in *Meiob*^−/−^ mice (Souquet et al. 2013; Luo et al. 2013). The immunoprecipitation of MEIOB from testis extracts revealed an interaction between MEIOB and RPA2, a subunit of the RPA complex, and SPATA22, a meiosis-specific factor essential to prophase I progression (Luo et al. 2013; La Salle et al. 2012). However, the role and the nature of these interactions remain to be determined.

### Putative roles of MEIOB

We identified *Meiob* as a meiosis-specific gene based on its differential expression between male and female embryonic germ cells at the time when only female germ cells have entered meiosis (Souquet et al. 2013). Luo et al. identified MEIOB through a systematical proteomic screen of meiosis-specific chromatin-associated proteins performed on mouse testes (Luo et al. 2013). The sterility of male and female *Meiob*^−/−^ mice is due to zygotene/pachytene arrest in prophase I of meiosis. MEIOB is essential to the repair of meiotic DSBs and to proceed through meiosis. MEIOB is located on chromosome axes from leptotene to pachytene (Souquet et al. 2013; Luo et al. 2013). The fact that MEIOB is recruited to meiotic chromosomes in a *Dmc1*^−/−^ mutant which is unable to undergo strand invasion of the homologous sequence suggests that MEIOB could play an early role on the ssDNA formed during DNA resection after DSB formation (Souquet et al. 2013). However, given that MEIOB persists until the pachytene stage, a later role in the processing of joint molecules formed during strand invasion is also possible (Souquet et al. 2013; Luo et al. 2013). Interestingly, we reported that in the absence of MEIOB, the recombinases DMC1 and RAD51 are loaded but not maintained on chromosome axes while RPA foci persist, suggesting that DSBs are not repaired (Souquet et al. 2013). This observation is reinforced by similar observations described in a rat mutant lacking functional SPATA22, a MEIOB partner (Ishishita et al. 2014). These results may suggest that the stabilization of the meiotic recombinases requires MEIOB and SPATA22. However, an alternative explanation would be that in absence of efficient DNA repair, the recombinases would be removed from recombination intermediates by helicase activity. The fact that RAD51 is loaded and not removed in a *Dmc1*^−/−^ mutant that is blocked prior to strand invasion, may suggest that recombination intermediates are not arrested at the same step in *Meiob*^−/−^ and *Dmc1*^−/−^ mutants (Pittman et al. 1998).

We found that only a fraction of MEIOB co-localizes with RPA2, particularly at early prophase I and, similarly to RPA, MEIOB is present on unsynapsed DNA (Souquet et al. 2013). These observations question whether MEIOB and RPA are loaded together on ssDNA formed on both sides of a DSB or individually loaded on each side of the DSB to provide different identities. We can also consider that similarly to AtRPA1c and AtRPA1a in *A. thaliana*, RPA1 and MEIOB could be involved in different steps of the meiotic recombination process. Interestingly, AtRPA1a is located on the chromosome axis from the leptotene to pachytene stage, similarly to MEIOB and RPA1 but is essential only after stabilization of the joint molecules, as suggested by the indistinguishable dynamics of MSH4 foci between wild-type and mutant. However, AtRPA1c is essential for meiotic recombination only in the absence of AtRPA1a, suggesting that AtRPA1a could overcome AtRPA1c early role during DSB repair. In contrast, RPA1 cannot overcome the absence of MEIOB, suggesting that MEIOB could possess activities similar to those of both AtRPA1c and AtRPA1a.

In vitro assays have indicated that the full-length protein, as well as a truncated form containing the second OB-fold, binds with higher affinity to ssDNA than to dsDNA (Souquet et al. 2013; Luo et al. 2013). These results confirm that MEIOB possesses ssDNA-binding activity, as predicted by the presence of the OB-fold. Strikingly, Luo et al. also observed Mg^2+^-dependent 3′-exonuclease activity specific to ssDNA when they expressed and purified the truncated form of MEIOB from *E. coli* (Luo et al. 2013). Such activity was unexpected for a paralog of RPA1, which is hypothesized to protect ssDNA from nucleases activities. Notably, no nuclease activity has been described for any RPA complex. To test the specificity of this activity, Luo et al. designed a mutation, S251A, which partially affected the exonuclease activity of the truncated MEIOB. However, the ssDNA-binding activity of the protein was also impaired by this mutation. Mutating acidic amino acids usually found in the catalytic site of nucleases should be of special interest because acidic amino acids are not thought to mediate interactions with nucleotides, unlike the hydroxyl function lost by mutation in a serine residue.

**Fig. 4:**
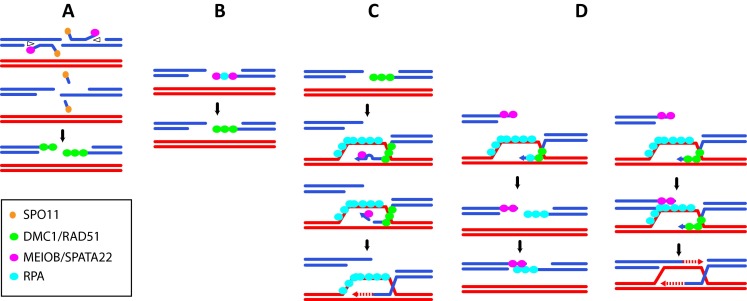
Putative roles for MEIOB during meiotic recombination. **a** MEIOB is loaded during the early steps of resection to release Spo11-oligo through its 3′-exonuclease activity with the help of a helicase (*opened triangle*). **b** MEIOB is loaded with or without RPA on the 3′-ssDNA tail. **c** MEIOB 3′-exonuclease activity removes the 3′ end of the invading strand in the presence of mismatches formed between the donor and invading strand to allow initiation of DNA synthesis. **d** MEIOB is loaded on one side of the broken end to allow strand annealing or second-end capture

Nonetheless, 3′-ssDNA tails need to be processed during meiotic homologous recombination. Luo et al. proposed that the putative exonuclease activity of MEIOB could process 3′ flaps formed after DNA synthesis during synthesis-dependent strand annealing (SDSA) or during dHJ formation. This activity may also be used after invasion of the homologous chromosomes, when sequence divergences between the invading strand and the donor induce the formation of DNA mismatches. Consequently the presence of these mismatches can induce the formation of a 3′ flap. In *S. cerevisiae*, it has been shown that during mitotic HR these 3′ flaps are processed by the endonuclease activity of the Rad1-Rad10 heterodimer to allow DNA synthesis to proceed and progress via recombination (Ivanov and Haber 1995). Through its nuclease activity, MEIOB may be involved in processing the 3′ flaps formed during strand invasion in meiosis (Fig. 4). Moreover, the MRE11 3′-exonuclease activity has been shown to be involved in the release of SPO11-oligonucleotides during meiotic DSB processing (Garcia et al. 2011). In a similar manner, MEIOB 3′-exonuclease activity with the help of a helicase could participate in early DSB processing (Fig. 4). Independently of its putative nuclease activity, Luo et al. proposed that due to its interaction with RPA2, MEIOB could provide a physical connection between an RPA-coated D-loop and the second broken end (Luo et al. 2013). An alternative possibility is that, similar to what has been proposed for AtRPA1a, MEIOB could be loaded on the second end and interact specifically with a RAD52-like factor and/or could possess an activity to anneal the second DSB end (Fig. 4). In addition, little is known about SPATA22. Similarly to MEIOB, SPATA22 is essential for meiotic HR and its invalidation induces a phenotype similar to that of MEIOB (La Salle et al. 2012; Ishishita et al. 2014). Moreover, the stabilities of MEIOB and SPATA22 are dependent on each other strongly suggesting that SPATA22 and MEIOB form a complex (Luo et al. 2013 and personal observations). MEIOB and SPATA22 show only a partial co-localization with RPA and are essential to meiotic HR. This observation suggests that MEIOB/SPATA22 and the RPA complex can act both independently and together on DNA. Deciphering the role of SPATA22 during meiosis will be essential to better understand the function of the MEIOB/SPATA22 complex during meiotic homologous recombination.

## Conclusion

In this review, we describe RPA-like proteins from different species. Studies in plants support the hypothesis that multiplying RPA family members may provide more specificity to the regulation of DNA metabolism and yield a larger panel of interacting factors. The existence of RPA4 in mammalian cells and MEIOB in metazoans and many fungi strongly suggests that this multiplicity is not restricted to plants. Because meiotic recombination is a conserved mechanism among species, we can investigate whether the role of MEIOB in species devoid of MEIOB could be supported by RPA. Conversely, MEIOB could also provide species-specific features to meiotic recombination. As an example, *S. cerevisiae* does not possess MEIOB. Interestingly, among the large panel of generated scRPA mutants and that affect specific functions, no meiosis-specific mutant has been identified (Soustelle et al. 2002). Based on the existence of homologies between scRPA and MEIOB which are absent in mammalian RPA, we also sought to identify specific meiotic domains in scRPA. Unfortunately, to date, the mutations of the putative domain tested did not allow us to identify any residues with a specific meiotic function in scRPA. These data would support the hypothesis that the presence of MEIOB provides species-specific features to the meiotic process, a hypothesis that will require further investigations. To better understand the role of MEIOB, it would be informative to know whether its recruitment is restricted to meiotic DSBs. Studying MEIOB localization during the repair of different types of DNA damage induced in mammalian mitotic cells after introducing *MEIOB* by transfection or in meiotic cells lacking meiotic DSBs (i.e., *Spo11*−/− mice) should shed light on the minimal requirements for in vivo MEIOB recruitment. Numerous uncertainties persist regarding the existence and putative purposes of different ssDNA-binding factors between mitotic and meiotic HR. Although RPA and MEIOB can partly co-localize on the meiotic chromosome axis, details of the dynamics of the various factors involved in ssDNA metabolism during meiosis are unclear. In this regard, deciphering whether MEIOB or RPA1 arrives and binds ssDNA first would be highly informative.

Understanding the nature and the role of specific ssDNA-interacting factors will provide essential key elements to improve our knowledge of the dynamics and regulation of meiotic recombination. This will also afford an original approach for deepening our understanding of the specific roles of canonical RPAs.

## Electronic supplementary material

Below is the link to the electronic supplementary material.

ESM 1 (DOCX 29kb)
