# Supplementary material for: RPA homologs and ssDNA processing during meiotic recombination
Source: Chromosoma. 2015 Oct 31;125:265–76. doi: 10.1007/s00412-015-0552-7 (PMC4830875; doi:10.1007/s00412-015-0552-7)
Supplement: Supplementary file 1 — (DOCX 29kb) [file 412_2015_552_MOESM1_ESM.docx]

>RPA1_Homo_sapiens (NP_002936.1)

MVGQLSEGAIAAIMQKGDTNIKPILQVINIRPITTGNSPPRYRLLMSDGLNTLSSFMLATQLNPLVEEEQ

LSSNCVCQIHRFIVNTLKDGRRVVILMELEVLKSAEAVGVKIGNPVPYNEGLGQPQVAPPAPAASPAASS

RPQPQNGSSGMGSTVSKAYGASKTFGKAAGPSLSHTSGGTQSKVVPIASLTPYQSKWTICARVTNKSQIR

TWSNSRGEGKLFSLELVDESGEIRATAFNEQVDKFFPLIEVNKVYYFSKGTLKIANKQFTAVKNDYEMTF

NNETSVMPCEDDHHLPTVQFDFTGIDDLENKSKDSLVDIIGICKSYEDATKITVRSNNREVAKRNIYLMD

TSGKVVTATLWGEDADKFDGSRQPVLAIKGARVSDFGGRSLSVLSSSTIIANPDIPEAYKLRGWFDAEGQ

ALDGVSISDLKSGGVGGSNTNWKTLYEVKSENLGQGDKPDYFSSVATVVYLRKENCMYQACPTQDCNKKV

IDQQNGLYRCEKCDTEFPNFKYRMILSVNIADFQENQWVTCFQESAEAILGQNAAYLGELKDKNEQAFEE

VFQNANFRSFIFRVRVKVETYNDESRIKATVMDVKPVDYREYGRRLVMSIRRSALM

>RPA1_Mus_musculus (EDL12800.1)

MVGHLSEGAIEVMIQQENTSIKPILQVINIRPISTGNRSPRYRLLMSDGLNTLSSFMLATQLNTLVEGGQ

LASNCVCQVHKFIVNTLKDGRKVVVLMDLEVMKSAEDVGLKIGNPVPYNEGYGQQQQQQQQQQQQAVPSP

ASAATPPASKPQPQNGSLGMGSTAAKAYGASKPFGKPAGTGLLQPSGGTQSKVVPIASLTPYQSKWTICA

RVTNKSQIRTWSNSRGEGKLFSLELVDESGEIRATAFNEQVDKFFPLIEVNKVYYFSKGALKIANKQFSA

VKNDYEMTFNNETSVLPCEDGHHLPTVQFDFTGIGDLESKAKDALVDIIGICKSYEDSIKITVKSNNREV

AKRNIYLMDMSGKVVTTTLWGEDADKFDGSRQPVMAIKGARVSDFGGRSLSVLSSSTVIVNPDIPEAYKL

RGWFDSEGQALDGVSISDHRSGGAGGGNTNWKTLHEAKSENLGQGDKADYFSTVAAVVFLRKENCMYQAC

PTQDCNKKVIDQQNGLYRCEKCDREFPNFKYRMILSANIADFQENQWVTCFQESAEAILGQNTMYLGELK

EKNEQAFEEVFQNANFRSFTFRIRVKLETYNDESRIKATVMDVKPVDFRDYGRRLIANIRKNM

>RPA1_Geospiza_fortis (XP_005425532.1)

MGLVIVRAIMQGEVVSKPVLQVINTRAIATGTGPPRYRVLMSDGVNTLSSFMLATQLNSLVEEERLSARC

VCQVNRFIVNSLKDGRRVVILMDVNVLQTADQVGGPIGNPQPYNEGQGQRSAAPAGNPAASKPQQQNGNL

SGAGPAAPKYHAPSNQFGKASAPSALKTPGGSQIKVVPIASLNPYQSKWTICARVTQKGQIRTWSNSRGE

GKLFSIELVDESGEIRATAFNDQADKFFPLIELNKVYYFTKGNLKTANKQYTAVKNDYEITFTNETSVVP

CDDAQHLPSVQFDFVSISDLENTPKDSIVDVIGICKSYEDVTKITVKANNREVSKRNVHLMDTSGKLVTA

TLWGNEAEQFDGSRQPVIAIKGARVSDFGGRSLSVLSSSTVVINPDSPEAFKLRGWFDSEGQLLECTSIS

DVRGGPAAGANTNWKTLFEAKSENLGQGDKADYFSCVGTIVHLRKENCMYQACPSQDCNKKVIDQQNGLY

RCEKCDREFPNFKYRLMLLVTIADCLEYQWVTCFQDTAEFILGQNAAFLGELKEKNEQAFEEVFQNANFN

TYEFRIRVKLETYNDESRIKATALDVKPVNYREYSKRLIANIRRNAQLG

>RPA1_Schizosaccharomyces_pombe (AAC49694.1)

MAERLSVGALRIINTSDASSFPPNPILQVLTVKELNSNPTSGAPKRYRVVLSDSINYAQSMLSTQLNHLV

AENKLQKGAFVQLTQFTVNVMKERKILIVLGLNVLTELGVMDKIGNPAGLETVDALRQQQNEQNNASAPR

TGISTSTNSFYGNNAAATAPAPPPMMKKPAAPNSLSTIIYPIEGLSPYQNKWTIRARVTNKSEVKHWHNQ

RGEGKLFSVNLLDESGEIRATGFNDQVDAFYDILQEGSVYYISRCRVNIAKKQYTNVQNEYELMFERDTE

IRKAEDQTAVPVAKFSFVSLQEVGDVAKDAVIDVIGVLQNVGPVQQITSRATSRGFDKRDITIVDQTGYE

MRVTLWGKTAIEFSVSEESILAFKGVKVNDFQGRSLSMLTSSTMSVDPDIQESHLLKGWYDGQGRGQEFA

KHSVISSTLSTTGRSAERKNIAEVQAEHLGMSETPDYFSLKGTIVYIRKKNVSYPACPAADCNKKVFDQG

GSWRCEKCNKEYDAPQYRYIITIAVGDHTGQLWLNVFDDVGKLIMHKTADELNDLQENDENAFMNCMAEA

CYMPYIFQCRAKQDNFKGEMRVRYTVMSINQMDWKEESKRLINFIESAQ

>RPA1_Saccharomyces_cerevisiae_S288c (NP_009404.1)

MSSVQLSRGDFHSIFTNKQRYDNPTGGVYQVYNTRKSDGANSNRKNLIMISDGIYHMKALLRNQAASKFQ

SMELQRGDIIRVIIAEPAIVRERKKYVLLVDDFELVQSRADMVNQTSTFLDNYFSEHPNETLKDEDITDS

GNVANQTNASNAGVPDMLHSNSNLNANERKFANENPNSQKTRPIFAIEQLSPYQNVWTIKARVSYKGEIK

TWHNQRGDGKLFNVNFLDTSGEIRATAFNDFATKFNEILQEGKVYYVSKAKLQPAKPQFTNLTHPYELNL

DRDTVIEECFDESNVPKTHFNFIKLDAIQNQEVNSNVDVLGIIQTINPHFELTSRAGKKFDRRDITIVDD

SGFSISVGLWNQQALDFNLPEGSVAAIKGVRVTDFGGKSLSMGFSSTLIPNPEIPEAYALKGWYDSKGRN

ANFITLKQEPGMGGQSAASLTKFIAQRITIARAQAENLGRSEKGDFFSVKAAISFLKVDNFAYPACSNEN

CNKKVLEQPDGTWRCEKCDTNNARPNWRYILTISIIDETNQLWLTLFDDQAKQLLGVDANTLMSLKEEDP

NEFTKITQSIQMNEYDFRIRAREDTYNDQSRIRYTVANLHSLNYRAEADYLADELSKALLA

>RPA1_Bos_taurus (NP_001068644.1)

MVGHLSEGAIAAIMQQGDTSIKPILQVINIRPITTGNSPPRYRLLMSDGLNTLSSFMLATQLNPLVEEER

LSSNCICQINRFIVNTLKDGRRVVILMELEVLKSAEAVGSKIGNPVPYNEGHGQQPVVPPPVSATSPPTS

RPQQQNGSPGMASTASKAFGASKTFGKAGGTSQVNSSGGTQAKVVPIASLTPYQSKWTICARVTNKSQIR

TWSNSRGEGKLFSIELVDESGEIRATAFNEQADKFFPLIDVNKVYYFSKGTLKIANKQFTAVKNDYEMTF

NNETSVMPCEDGHHLPTVQFDFTGIGDLESKSKDSLVDIIGICKNYEDVTKIIVKSNNREVSKRNIYLMD

MSGKVVNATLWGDDADKFDGSRQPVMAIKGARVSDFGGRSLSVLSSSTIIVNPDIPEAYKLRGWFDSEGQ

ALDGISISDLKSGGAGGSNTNWKTLYEVKSENLGQGDKPDYFSSVATVVYLRKENCMYQACPTQDCNKKV

IDQQNGLYRCEKCDSEFPNFKYRMILSVNIADFQENQWVTCFQESAEAILGQSTAYLGELKEKNEQAFEE

VFQNANFRSFTFRIRVKLETYNDESRIKATVVDVKPVDYREYGRRLVMNIRRNAAM

>AtRPA1A (Q9SKI4)

MPVLEIKMIGRSQERSQERYRFLISDGVSAQHAMVAVQLNDRVKSGQFEKGSIVQLIDYI

CSDVKGRKLIVVLNMETIVQQSETIGNPTIFGETDTEAQKTFSGTGNIPPPNRVVFNEPM

VQHSVNRAPPRGVNIQNQANNTPSFRPSVQPSYQPPASYRNHGPIMKNEAPARVIPIAAL

NPYQGRWAIKARVTAKGDIRRYNNAKGDGKVFSFDLLDYDGGEIRVTCFNALVDRFYDVT

EVGKVYLISKGSLKPAQKNFNHLKNEWEIFLESTSTVELCPDEDGSIPKQQFSFRPISDI

ENAENNTILDVIGVVTSVNPSVPILRKNGMETHRRILNLKDESGKAVEVTLWGEFCNRDG

RQLEEMVDSAFHPVLAIKAGKVSDFSGKSVGTISSTQLFINPDFPEAHKLRTWFDYGGKD

TASFSISRDTMPGGVSRNEIRKNVSQIKEEGLGRSDKPDWITVKATISFIKTDSFCYTAC

PLMIGDKQCNKKVTRSGTNRWLCDRCNQESDECDYRYLLQVQIQDHTGLTWITAFQETGE

EIMGCPAKKLYAMKYELEKEEEFAEIVRDRLFHQYMLKLKIKEESYGDEQRVKMTVVKVD

KVNYTSESKYMLDLLVR

>AtRPA1B (Q9SD82)

MENSVTQDGIATVLANQSLDSSSVRPEIVVQVVDLKPAGNRYTFSANDGKMKIKAMLPAT

LTSDIISGKIQNLGLIRLLEYTVNDIPGKSEEKYMLITKCEAVASALDSEIKAEIKASTG

IMLKPKHEFVAKSASQIINEQRGNAAPAARMAMTRRVHPLVSLNPYQGSWTIKVRVTNKG

VMRTYKNARGEGCVFNVELTDEEGTQIQATMFNAAARKFYDRFEMGKVYYISRGSLKLAN

KQFKTVQNDYEMTLNENSEVEEASNEEMFTPETKFNFVPIDELGTYVNQKDLIDVIGVVQ

SVSPTMSIRRKNDNEMIPKRDITLADETKKTVVVSLWNDLATGIGQELLDMADNHPVIAI

KSLKVGAFQGVSLSTISRSNVVINPNSPEATKLKSWYDAEGKETSMSAIGSGMSSSANNG

SRSMYSDRVFLSHITSNPSLGEEKPVFFSTRAYISFIKPDQTMWYRACKTCNKKVTEAMD

SGYWCESCQKKDQECSLRYIMAVKVSDSTGETWLSAFNDEAEKIIGCTADDLNDLKSEEG

EVNEFQTKLKEATWSSHLFRISVSQQEYNSEKRQRITVRGVSPIDFAAETRLLLQDISKN

KTSQ

>AtRPA1C (Q9FHJ6)

MAVSLTEGVVMKMLNGEVTSETDMMPVLQVTELKLIQSKLHQNQESSNRYKFLLSDGTDL

AAGMLNTSLNSLVNQGTIQLGSVIRLTHYICNLIQTRRIVVIMQLEVIVEKCNIIGNPKE

PGHSSINPQRGGVNTQSNGGSEQQQARRSDVNGGRYGVSANSPQPQVVHNSSDAGRYCVS

ANSPQPQVVHSSSDAGRYGVSANSPQRQVVHNSPDAGRYGQPQVSQRYGTGSGYPETSPS

TRPYVSSNAGYGGSRQDQPRAPTATTAYSRPVQSAYQPQQPPMYVNRGPVARNEAPPRIN

PIAALNPYQGRWTIKVRVTSKADLRRFNNPRGEGKLFSFDLLDADGGEIRVTCFNDAVDQ

FFDKIVVGNVYLISRGNLKPAQKNFNHLPNDYEIHLDSASTIQPCEDDGTIPRYHFHFRN

IGDIENMENNSTTDVIGIVSSISPTVAIMRKNLTEVQKRSLQLKDMSGRSVEVTMWGNFC

NAEGQKLQNLCDSGVFPVLALKAGRIGEFNGKQVSTIGASQFFIEPDFPEARELRQWYER

EGRNAHFTSISREFSGVGRQEVRKVIAQIKDEKLGTSEKPDWITVCATISFMKVENFCYT

ACPIMNGDRPCSKKVTNNGDGTWRCEKCDKCVDECDYRYILQIQLQDHTDLTWATAFQEA

GEEIMGMSAKDLYYVKYENQDEEKFEDIIRSVAFTKYIFKLKIKEETYSDEQRVKATVVK

AEKLNYSSNTRFMLEAIDKLKIGDANSLPIKAESSNYRSDAFNSGVGTSGTRDTASVDAR

REFGLPAANQVGQYGNQYSSDARSLGGFTSCNVCRSNSHVSANCPTLMSEPQGQYMGGTN

AGGGMPRQHVGSY

>AtRPA1D (Q9FME0)

MQTSVTPDAISTVLSNPSFDSSSDRSEIVVQVVDLKPIGNRYTFSANDGKTKVKAMFTAS

LTPEIISGKIQNLGLIRLIDFTVNDISSKSTKYFLVTKCEAVGSVLDSEINLDSKSGEEE

AREPKKQKLEHSPVSPLNDVVSTGITLKPKQEFVAKSASQIMSEQRGNAAPAARMAMTRR

VHPLVSLNPYQGNWTIKVRVTNKGVMRNYKNARGEGCVFNVELTDEEGTQIQATMFNDAA

RKFFDRFQLGKVYYISRGSLKLANKQFKTVQNDYEMTLNENSEVEEASSEEMFIPETKFN

FVPIEELGLYVNQKELIDLIGVVQSVSPTMSIRRRTDNEMIPKRDITLADESRKTVVVSL

WNDLATGIGQELLDMADQSPVIAIKSLKVGDFQGVSLSTISRSNVVINPESPEAKKLKSW

FDSEGKEISMSSIGSGMSPSAKNGSRSLYTDRVLLSHITSNPSLFEEKPVFFSTRAYISF

IKPDQTMWYQACKTCNKKVTEALDSGYWCEGCQRKYEECSLRYIMAVKVTDSSGETWISS

FNDEAEKILGCSADELNKLKSEEGEVNEYQTKLKEATWSSHVFRVSVTQNEYNGEKRQRV

TVKGVAPLDFAAETRLLLQDISNKNKTSQ

>AtRPA1E (F4JSG3)

MEVSLTAGAIGKIMNGEVTTEADMIPVLQVTDLKQIMAQQDPTRERFRMVLSDGTYLHQG

MLGTDLNNLVKEGTLQPGSIVRLTRFVGDVIKGRRIVIVPQLEVLKQISDIIGHPVPGGK

HNDQRGADSGIKFNTTEQQGSGIRQVNNIEPGRSNAAISPQVGGTGSSVPASTTPSTRAY

SNPSSGNGVTRQDYARDPPTSYPHQPQPPPPMYANRGPVARNEAPPKIIPVNALSPYSGR

WTIKARVTNKAALKQYSNPRGEGKVFNFDLLDADGGEIRVTCFNAVADQFYDQIVVGNLY

LISRGSLRPAQKNFNHLRNDYEIMLDNASTIKQCYEEDAAIPRHQFHFRTIGDIESMENN

CIVDVIGIVSSISPTVTITRKNGTATPKRSLQLKDMSGRSVEVTMWGDFCNAEGQRLQSL

CDSGVFPVLAVKAGRISEFNGKTVSTIGSSQLFIDPDFVEAEKLKNWFEREGKSVPCISL

SREFSGSGKVDVRKTISQIKDEKLGTSEKPDWITVSATILYLKFDNFCYTACPIMNGDRP

CSKKVTDNGDGTWRCEKCDKSVDECDYRYILQLQIQDHTDLTCVTAFQEAGEEIMGISAK

DLYYVKNEHKDEEKFEDIIRKVAFTKYNFKLKVKEETFSDEQRVKATVVKVDKLNYSADT

RTMLGAMDKLRTRDANSLPINPEGSDYNADVVNTGIGSSGTRDPSSVQRRDFGLHAHQSG

QSGNHYSGGGATTSCNVCGNSGHVSAKCPGATKPQEQGQYMGGSYRGTTGSYGGGLPRQH

VGSY

>OsRPA1A (Q6YZ49)

MAMARLTPNGVAAALAGDTNLKPVLQIVELRGVQVNGAGVTRGERFRAVVSDGTAASSAL

FAAQLSDHARSGALRRGSIVQLSEYVINEVGPRRIIVILNLEVLVSECEIIGNPTALSET

GSPIPNPTRVEQFNGAPQYGLMAGNSSNTTTKPSDNVPLFQNSMAGNSSNFATRPSDKVP

VFQPTVQPSYRPAPNYKNHGAIMKNEAPARIIPISALNPYQGRWAIKARVTAKGDIRRYH

NAKGDGKVFSFDLLDSDGGEIRVTCFNALLDRFYEVVEVGKVYVVSRGNLRPAQKNYNHL

NNEWEILLENGSTVDLCPDENSSIPTQRFDFRPINEIEDAQNNAILDIIGVVTSVNPCTT

IQRKNGMETQKRTMNLKDMSGRSVEVTMWGDFCNREGSQLQGMVERGIFPVLAVKAGKVS

DFSGKSVGTISSTQLFINPDSAEAHSLRQWFDSGGRDASTQSISRDITPGASRNEIRKTV

AQIKDEGLGMGDKPDWITVKATVIFFKNESFFYTACPNMIGDRQCNKKVTKSTNGNWTCD

KCDREFEECDYRYLLQFQIQDHSGTAWVTAFQEAGQELLGCSATELNALKEREDPRFADT

MLNCLFQEYLLRLKVKEESYGDERKVKNTAVKVEKVDPSGESKFLLDLISKSSALH

>OsRPA1B (Q10Q08)

MDSDAAPSVTPGAVAFVLENASPDAATGVPVPEIVLQVVDLKPIGTRFTFLASDGKDKIK

TMLLTQLAPEVRSGNIQNLGVIRVLDYTCNTIGEKQEKVLIITKLEVVFKALDSEIKCEA

EKQEEKPAILLSPKEESVVLSKPTNAPPLPPVVLKPKQEVKSASQIVNEQRGNAAPAARL

AMTRRVHPLISLNPYQGNWIIKVRVTSKGNLRTYKNARGEGCVFNVELTDVDGTQIQATM

FNEAAKKFYPMFELGKVYYISKGSLRVANKQFKTVHNDYEMTLNENAVVEEAEGETFIPQ

IQYNFVKIDQLGPYVGGRELVDVIGVVQSVSPTLSVRRKIDNETIPKRDIVVADDSSKTV

TISLWNDLATTTGQELLDMVDSAPIIAIKSLKVSDFQGLSLSTVGRSTIVVNPDLPEAEQ

LRAWYDSEGKGTSMASIGSDMGASRVGGARSMYSDRVFLSHITSDPNLGQDKPVFFSLNA

YISLIKPDQTMWYRACKTCNKKVTEAIGSGYWCEGCQKNDAECSLRYIMVIKVSDPTGEA

WLSLFNDQAERIVGCSADELDRIRKEEGDDSYLLKLKEATWVPHLFRVSVTQNEYMNEKR

QRITVRSEAPVDHAAEAKYMLEEIAKLTGC

>OsRPA1C (Q65XV7)

MEPQLTPGAVQAIAEHPDGTGTIQPVLQVVDVRPVTTKNAPPTPKPAERFRMMLSDGVNT

QQSMLATALNPLVKDATLRPGTVVQLTDFMCNTIQGKRIIIVVKLDVLQNDCIVIGNPKH

YEPKSLTKEQDPNLQASVAQTNNGTYSGGASMLGPSVAPRAEQAASNSSYGGPYNSAQGM

LGSSIGRTVEPGPANVSAVGSYGAISAQNTTNANMMQPTSQLNIMNANTMQPTSQLNTMN

ANTMQPTSQLSSLNPNQNQRFAAPASGGVFGPPGNAYGQPSRPSYQQPPPVYMNRGPASR

NDSATRIIPITALNPYQPKWTIKARVTAKSDIRHWSNARSSGTVFSFDLLDAQGGEIRAQ

CWKESADKFFGQIEVGRVYLISRGSLKPAQKKYNTLNHDYEITLDIGLSTVEVCSDDDNS

IPRLQYNFRQISELENMANETIVDLLGVVTSVSPSATIMRKIGTETRKRSIQLKDLSGRS

IEVTLWGNFCDAEGQQLQLQCDSGSNPIIAFKGARVGDFNGKSVSTIGSTQLIINPDFPE

VERLRQWYMTEGKTAPCISLSREMLNMGRTDARKTIAQIKDENLGRLEKPDWITVKAAIS

HVTTESFCYPACPKLLPVGRQCNKKAINNGDGMWHCDRCDESFQNPEYRYMLRFQIQDHT

GSTYASAFDEAGEQIFGRKAGELFSIRNVDQDDAQFAEIIEGVRWHLYLFKLKVKEETYN

DEQSLKCTAVKVEKLDPSKESNVLLGAIDNLLLDPKGQSDLAPNAGFTDPVGGHGAPTSS

NAYAMNTGGVNQFGQQASISAGMSTPLAATRNLQTCSICGANGHSAQICHVGADMDMQET

SAGGSSMGNYNSIAGNGSSECYKCKQPGHYARDCPGQSTGGLECFKCKQPGHFSRDCPVQ

STGGSECFKCKQPGHFARDCPGQSTGAQHQTYGNNVAASRGYNRQSFVGGY

>RPA1_Mortierella_verticillata (KFH74008.1)

MTLHPTAGAIKAIFNEEAGNPAANAPILQVLNTKMLPSTSGAAANRYRTMFSDGVHVMQGIFATTLNNLI

DSGAITKNSLVRVNRYTIKPLAGKKILLVVEVDVLDNVGGTYEKFGDPVHIDPEVHNQPNQGRPAQQSSY

QQQQHQQQQRNNPFQQQQNPYQQNAHQPQINQNHQPQIAPSGNPVFPITALNPYQNKWTIMARVTQKSDI

RTWSKPGGNEGKLFSMTLTDESGEIKVTGFNQQVDDYFNVVEQDKVYFLSNAKVDFAKKQFSNVKNEYEL

ILQRDSQLIPAPDNAHVPAVRYNFVNLTNLSNLNEKDTVDIIAIIKEVGEFTNNISQKTNKPLLKRDLTL

VDASGMSTRLTLWGATAESFNPGSANPVIAFKGVSVSNYGGKSLNAFGGSTFKLNPEINEAFELRGWFDQ

QGHNTNFQSHTSDFKAGGTAPRMTVDEFTVAAANLNPNEQLNFEVKASIVMMAKAETTFHYPACPTKGCN

KKVTEDMNQQWRCEKCNQSFPEPDYRYVMRANIGDQSGTAWVQAFNEAGEVITGKKAYDLVTRPGEVKPT

FDQANFKSYIFRCRAKQEVFNDEAKIRYAIIDITPVNWVEESKLLLSKLKEFGI

>RPA1_Neurospora_crassa (KHE88815.1)

MAGQITQGALDAMFNDPDRAQQQFPVPILQCLQIKTLDSKNGGAGATERFRIVLSDLKNYVQCMMATQTN

HLVHDGLLQRGCIVRLKQYQAQCLKGKNILIVLDLEVIQSLGCPEKMGDPQPLGPRSAEPQQNPNLGSTG

FYGVKSEPTQDTKPQFPRQMPSRNASGGQGSSTIYPIEGLSPFSHKWTIKARVTSKSDIKTWHKASGEGK

LFSVNFLDESGEIRATGFNDQVDQFYDLLQEGQVYYISTPCRVQLAKKQWSNLPNDYELTFERDTVIEKA

EDQTSVPQVRFNFVNIQELQDVERDATVDIIGVLKEVQEVTQIVSKTTQKPYDKRELTLVDNTGYSVRCT

IWGKTATNFDAQPESIVAFKGTKVSDFGGRSLSLLSSGTMAIDPDIPEAHHLKGWYDSSGRNNTFATHNN

MQTLGGATGRKDDAKTISQVKEENLGTNEAPDYFALKATVVFIKQDNFAYPGCRSEGCNRKVTDMGDGTW

RCEKCQINHDRPQYRYIMSVNVNDHTGQLWLSCFDDTARVIMGKSADELMEIRETDETRLPAEFEQANCR

KLNFRCRAKMDTFGEQQRIRYQVMSVAPLDYKMEGNKLNELINSYNQMSM

>RPA1_Coniosporium_apollinis (EON65480.1)

MSAEAASHITQGALRAVFEQGREAVGEPVVQAVQIKPLEAKGDGPERYRVVFSDMQNFVQSMIAEKYSSV

VSDGTLKRGAIVRLIQYTPNVIKGKKILIVGEVQVLSELGEHEKLGNPKGLEAAPEQEQQNAQPGSISGD

GFYGAKPQQQQLQPQRQNLPSRTNVSADSSANHGNIYPIEALSPYSHKWTIKARVTYKSPIKTWHKQSSE

GKLFSVNLLDESGEIKMTGFNAECDAWYNVLQEGSVYYISSPCKVNMAKKQFSNLNNDYELALERDTVIE

AAPDQSSVPQVRFNFTTIGDLESVEKNTTIDTIGVLQQVGEVSEIVSKTTSKPYAKRELTLVDNTNHSVR

LTIWGDTAHSFDAPEGSAIAFKGVKVSDFGGRSLSLLSSGSMTIDPDIDEAHKLKGWYDAQGRQDTFQSF

QNMGASLGGGGNSSKTIQEVKDENLGMNEEKPDYFNLKATVVFIKQDNIAYPACLSPDCNKKVVEVDPGQ

WRCEKCDKTHPRPQYRYMMSINVSDHTGQMWLSAFDDHGRLLLGMDADQIMAWKEEENKQALDDAFQAAN

CKTYVFRCRAKMDNFQDQQRVNLTTQKRLAASVAGCGKRKIWMDPNESNELSNANSRNTIRKLLKDGLII

KKPVTMHSRARARALTAARRIGRHRGFGKRKGTADARMPSQVLWMRHQRVLRRLLVKYRASGKIDKHLYH

ELYALSKGNTFKHKRALVEHIHRAKAEKQRERIIKEEMDAKRAKTRAARERRQQRVEEKRQATVAEE

>RPA1_Pneumocystis_murina (XP_007872715.1)

MTEPISIGSLKPILNSQPGALQTPIFQVLQVRKLTSVSNGVERYRIVLSDSIHYVQSMLASQKNELVVSG

KLQKGTIVQLIQFSVNMMKERKIIIIINLDVLEQYGVLPKIGTPASLEASSTTADKEEDHKQEIPVINQT

SFYGNKPLEPTFHGQKGKMNHISSVSSTTIYPIESLSPYQNKWTIKARVTNKSEIKHWHNQKGEGKLFSC

IFMDESGEIRATAFNDQVDMLYDVLQEGQVYFVSKCRVNIAKKQFSNVQNEYELTFEKDTEVERCPDQSS

VPHVKFNFVSLKDLDSVEKDSIIDVIGILNDIHESVEITSKTTQKLYSKRDVFLVDNSNYSVRLTLWGKH

AQDFNIPQETIVAFKGLKVSDFNGRSLSMLNSGMIIADPQIEEAYFLKKWYDKQGKEETFITHQPTITTI

RKEERKTIAQIKDEQLGMTEQPDYFSVKATIVFFKQENIFYPACPIEGCNKKVIEDNEGRWRCERCDKSF

LKPQYRYIMTISVNDHTGQIWLNCFDDVSRQIIGKSADDIVQIKEENEQSALNIFHEANCKSYVFRVRAK

QDSYNGAVRVRYQVMGVSPINWAYECKLLAEIINSY

>RPA1_Capsaspora_owczarzaki (XP_004363840.1)

MAEFTLTAGAIEAMSNPAKASNYVVQPVLQILSIKKLTSNGTTPGDRHRVVISDGVHHYMQALLGTSLAE

VAKTDAITRFTVVRVKKFVCSPVVERTIVILMDVDILGTPAGKIGDPKPYEVAPDAGAVAGGAAAGAPTS

PAGATNGAAAPVGAAPAAPKVSPYRQYTDAATGAVTSAFGARVASPPRAQQSFPAFDPNKTLEQIVAEGQ

TPETLPIAYIYNTMPSKFIIRARVTLKHPVREFARKNPGPNNQSGGRVCSIDLKDDSSEIRATAFNEQID

TIINSLEVGKAYYFSKMSVKPANRMYNTLPSDYELTFEKGTQWVPCHDEANLPQVVYNFVRFDGLETLGE

QTVDLIGICKSARDVQTITTKQQKSVPKRELTLVDQSQREITVTLWNTQATNFDEQVAVDNRVLAFRKVK

LTDFSGVKSASCISSSAMEVEPDMPETQELRAWFDSEGRNQSFQQVTGGAGAYSGGNRDLATFQQINSNH

SLGADKAEYARLQGTVTFLKTKFDDKSGSNDGSVGMYYYACGNIKQPIDNGQAAAPNSRQQAQQPSVCGK

KLAIGEQCPTCGDVSQRISGCNLTFVADDSTGSQWITAFRDTAQTILGASNKDLTLEHLHELALNGEQEQ

LDSIYAQALHHPYEMTLRIKQEQSQQGGMRTRYQLVRLAPIDFARDSLRLVRLIQAYN

>RPA1_Drosophila_melanogaster (AAF54206.1)

MVLASLSTGVIARIMHGEVVDAPVLQILAIKKINSAADSERYRILISDGKYFNSYAMLASQLNVMQHNGE

LEEFTIVQLDKYVTSLVGKDGAGKRVLIISELTVVNPGAEVKSKIGEPVTYENAAKQDLAPKPAVTSNSK

PIAKKEPSHNNNNNIVMNSSINSGMTHPISSLSPYQNKWVIKARVTSKSGIRTWSNARGEGKLFSMDLMD

ESGEIRATAFKEQCDKFYDLIQVDSVYYISKCQLKPANKQYSSLNNAYEMTFSGETVVQLCEDTDDDPIP

EIKYNLVPISDVSGMENKAAVDTIGICKEVGELQSFVARTTNKEFKKRDITLVDMSNSAISLTLWGDDAV

NFDGHVQPVILVKGTRINEFNGGKSLSLGGGSIMKINPDIPEAHKLRGWFDNGGGDSVANMVSARTGGGS

FSTEWMTLKDARARNLGSGDKPDYFQCKAVVHIVKQENAFYRACPQSDCNKKVVDEGNDQFRCEKCNALF

PNFKYRLLINMSIGDWTSNRWVSSFNEVGEQLLGHTSQEVGEALENDPAKAEQIFSALNFTSHIFKLRCK

NEVYGDMTRNKLTVQSVAPINHKEYNKHLLKELQELTGIGSSN

>MEIOB_Homo_sapiens (NP_001157032.1)

MANSFAARIFTTLSDLQTNMANLKVIGIVIGKTDVKGFPDRKNIGSERYTFSFTIRDSPAHFVNAASWGN

EDYIKSLSDSFRVGDCVIIENPLIQRKEIEREEKFSPATPSNCKLLLSENHSTVKVCSSYEVDTKLLSLI

HLPVKESHDYYSLGDIVANGHSLNGRIINVLAAVKSVGEPKYFTTSDRRKGQRCEVRLYDETESSFAMTC

WDNESILLAQSWMPRETVIFASDVRINFDKFRNCMTATVISKTIITTNPDIPEANILLNFIRENKETNVL

DDEIDSYFKESINLSTIVDVYTVEQLKGKALKNEGKADPSYGILYAYISTLNIDDETTKVVRNRCSSCGY

IVNEASNMCTTCNKNSLDFKSVFLSFHVLIDLTDHTGTLHSCSLTGSVAEETLGCTVHEFLAMTDEQKTA

LKWQFLLERSKIYLKFVLSHRARSGLKISVLSCKLADPTEASRNLSGQKHV

>MEIOB_Mus_musculus (NP_083473.1)

MAKFFALKNFTALSDLHPNMANLKIIGIVIGKTDVKGFPDRKNIGSERYTFSFTIRDSPCHFVNVSSWGS

EDYIRSLSESFRVAECVIIENPLIQRKDTEREEKFSPATPSNYKLLLSENHSMVKVCSPYEVDTKLLSLI

HLPVKESRDYYSLADIVANGHSLDGRIINVLAAVRSVGEPKYFTTSDRRKGQRCEVKLFDETEPSFTMTC

WDNESILLAQSWMARETVIFASDVRINFNKFQNCMAATVISKTIITVNPDTPEANILLNYIRENKETNVA

DEIDSYLKESVNLNTIVDVYTVEQLKVKALKSEGKADPFYGILYAYISTLNIDDETTKVVRNRCSSCGYI

VNEASNTCTICNQDSSRLKSFFLSFDVLVDLTDHTGTLHSCSLSGSIAEETLGCTINEFLTMTSEQKTKL

KWQLLLERSKIYLKLILSHRARGGLKVTILSCKLADPTEASRNLARQGHT

>MEIOB_Bos_taurus (XP_005196960.1)

MANSIASKNFTALSNLHPNMANLKIIGIVIGKTDVKGFPDRKNIGSERYTFSFTIRDSTTHFVNATSWGS

EDYIRSLSDSFRVGECVIIENPLIQTKDLEREEKFSPATPSNYKLLLSENHSTVKLCSSYEVDAKLLSLI

YLPVKESCDYYSLGDIVANGHSLDGKIINVLAAVRSVGEPKYFTTSDRRKGQRCEVKLYDETESSFAMIC

WDSESILLAQSWVPQETVIFAADVRISFDKFRNSMTATVISKTIITTNPDTPEANILLNFIRENKVMNPL

DDEIDNYLKESMNLNTIVDVYTVEQLKVKALKNEGKAEPFYGILYAYISTLNIDDETTKVVRNRCSVCGY

TVNEASSTCTTCNKDSSGFKSVFLSFDMLIDLTDHTGTLHSCSLTGSVAEETLGCTVDEFLAMTDEQKTA

LKWQFLLERSKIYLKFFISHRARGGLRVSVLSCKLADSIEASRNLSGQGSVQN

>MEIOB_Geospiza_fortis (XP_005426058.1)

MAHSRSARDFVALSDLHPNLARPNVIGLVIGKTDVRSFPDRKNIGTERYTFSFTIRDSPTYFINVQSWGR

EEYIRSLSESFRVGDCVTIENPLIQTKEAEREEKFNPVTPSGYKLLLSENHSVVKTSSCYDTDTRLLALL

HLPVKDPQDYYSLGDIVANGQSLHGRVLNVLAAVMAVGEPKYFMTSDKRKGQRCEVKLYDETERSFPIVC

WDNESIQLAQSWIPQETVIFASDVRINFDKFRNCMTATVISKTIITTNPETAEANVLFSFIKESAQAGAL

PSPVKELPNETINLEAVVDVYTVEQLKEKALQSDGKLEPVHGIIYGYISTLDIDESVSRVLRNRCSVCRF

IVNEASNTCTFCTDVSPEAKSTFASFDILVDVTDHTGTLRSCYLADCVAEDTLGCTVPEFLMLEEDQKTA

LKWQLLLERSKIYFKVTSSPNWRTGLKVNLLSCKLADPIEASQSLLGRDWNYL

>MEIOB-like_Morietella_verticillata (KFH68949.1)

MYRNQHPARPVFSGSMENLAHQAQQRHQYTSHQPYHNTCIQQMAPHGSLTQGTPPYRNYSPVPGPFPTFS

PQALLTFAPRQLGVTNDAPMVPIQSLRPEKAFVRVSGRTVRPPTVKAMPDKYESNKVRWLQSFAIKDDLD

TIEVAFWHNTSEHLEMNSGITLDQVVHVWTDDVKPKSKPSFGNSSPSTSSPLMLTLSEGKAGHKVVPGND

QEQATMFRTALGVNSGGIVDMVYIRNIVDAVKIPEHQRRNVIVCVKKLNPASITNTKNGPKARRVITVFD

SKGQDAVLTLWGDEMCSTADSWISMETGAQLSMYAMKPQISVSFQTHIQVNPICKNVEWLRHFTDQCASL

PDQLNPVADPNEVQLTQIQSSYHIIDISSCLDTLGFLETVYGFSYAVICQLDIDNHRDILTSKCPSCKKS

ITSFKIEACPSCRVVPPNENQWQYNMSRYISFMDHTAELTHPNVPSSIISELFGFQPKDFGRLSMVERAQ

LKRRFFLERFKIYFKIANSSTGSQPRVQILSIERAHLPEVVA

>MEIOB-like_Neurospora_crassa (CAD70895.1)

MVIPPIQSFYQKASPASSAFSQTLPGTTISNHADMSRAPLTETWKPQGSYARVNISELQPGRGKVRFMGR

IVNICPAKPDHQPRALSSLPSGFHFMVVKDDTGVVAIKLLGTQSDLINLHLGNLVTVWTGFVAEYSTAAT

IQVPFVSMIIPVHPGPASQSCIKFHQNGMNPEETGLCRIPLEYNTSSASSQMPGLISLRAFMKGTHTKGK

HEDDILTNILVCVSSIGPRKTIKANNKPSTLELVEVHVCDETDHCVLKLWSDQIVSAREWTAGQTILLIT

NPKLYPRKKGNENPGLGVALNSIVDVDPDFPDAHWLRRMAEAQRKRERAHTPFPVGVWDVEQAINGPNRV

LYTLADVDERVREDASVVFTGKLNVLILGVSICESQRQNRLCCFEWPYSQKHRRCARYPWYSFCRSLLEH

GGSFPQVSWATELLVVFVAFIDG

>HDM_drosphila_melanogaster (Q9W3M9)

MARRIKFQRLAEMRPTMTRFSTVALIVSKSSPNIFYDKMSGTERGVLSLTIRDSPNHLTN

CKCWGQRDCVDEYAAMLQIGHVVDIVGAKVMSIPFAAPGEQRYQPQATVSCALVVNEGSG

YVVRHDNDDFGQITILQQLLHQPIRPLGAVLKLADVRSGLGFPDKIITTNVNLLVVVAAV

RPVRQIKRKLQGPLSEVQELLQCLEVIVIDASYPEGMLLSVWQPDWIQRAQQWQPRRTVL

HLIDVRVSYSNFHRSLVLSHSNCTLICENPQAAGDDCRLLLAFAATVPLTTFSGCDQAEL

DNMPAVASIQAQMTVRQIYSRAEGELQDPSIHQFTAVLYGMVTKFDLDGLTSHVNRKCIA

CQQHIPRNLQDCASDACQQYFSLDNDEPRSISYFNINIHLSDQTGTLVEARLAGHPAERI

LGLRAEDFERLAEREKSELKWRFLLKYFEVRLMIKKPVGVRNHLVIVVVDMQAIPLEKLV

ANMVVF

>MEIOB-like_Coniosporium_apollinis (XP_007785052.1)

MAPQFPSIEQFFEKQSSQSPKKRARSSSPSQGRDGFTEAEVDAVLHPPANQTWSPPNEYEEIDIAALIPG

PKCVTFVGRVANFYDQGTPSKKPRAAKGCVKIIVKDDTGALTVRLWYANKDYNLRLGHLVTVWTPHISHS

DTGQLAPSSAPLFASIFPERDRSCHFMVHENSDEGVQCKTPLGYKVGQPLAGLMTLKNFIEGGYEITDGK

ILVCVKSIGPRKKFTTKKGIPAENVNIGVFDDTAEASLTLWGSSAASAGAWKVSHTILLITEPGWRIDKR

AWISLTSSTQVDVNPAILDTEWLRGFAQRLTRREHVNPPFPEGVFDTEAAEESQVRILYTLADIDEFART

APGGKSSFGHQKFMGYLSVIIMELNITSLHRRNMLMCTECCGVPLYANATSAKCKQCDKEIILRINPRLL

GPLIDETGTTSTGKLILSAAAWKQLLGRTAQELVASAADILKYLEQRLLFVRVTLLFGWIAEEGEGVGRL

CIWGVRM

>MEIOB-like_Pneumocystis_murina (XP_007871920.1)

MEVFPCEKAFYKNIFEKNTSFDDKWEAGNGFEPNNLYSKHSEIYQDNWYPKIKYEETKIKELSESNSPIA

LTGRIVNIYEINNQKNIENTLILIYIKDETDVVEVKYWLNKRNVKEIHYNLKIDTLCTAFVTKTCIKKED

ISRFKKASTVPFYVIVSDDNEITHLSIFKNQDKIYKKPLKIQKDCIIQDLMSLSTFINGGYMAISKPKVL

VSIRKIGNIRKINTKNGYSDMIELRVFDDTYDAVLILWGNNAKSAELWTPFDTILLLTDVSLSFRCEKAY

LTFQKYSIVEVNPDMDIEWLRKYSTDISNKVSDMHKLSLEDRIISTSPTMATYTLAEIDTMIRLNPKEPI

IGFINVLIVKTAKLSTLRSSNKIFVGKCCDKVINALDNNVKCSICHQKIKLFNDSFIIGLLDESAQLLYP

MLNLYNSSNSELDDFSDTILYSRILFLFGIERNDDIWKVNLIKIVDNIK

>MEIOB_Capsaspora_owczarzaki (XP_004346178.1)

MSRATIDQLSAGMQSLTVVGLVVAKLSIGTFEDRKNPGTSRSRFTFTLRDSESDSINCTMWGDPNLIGSA

HSRFNTGDVVEVIHPQVVPKNPTQEQRWMPRTSSAYALQLNSSSDVQLYMGDLPDRQRFVRIPMLYESGF

VKIQDILASPQELSQQYINVLGIVSRIGAVTSTTTSNGRATDKLEVYLTDDSGGEIRMTLWGSCIAMASN

WTVRDTILFLSDVRVRTSSFVPSAARNDPTQEHERMVYTTELSFDNCSLVTEYPDLREAQELYDYAQQLP

ADAFTTASRMGGGMSRAAFKPIRNDEIVESQGKFVNVDVAGVYALRPPVFVFDVFITDLPLDDFYTQVVI

ARCPGCNQQLPVEHACNRPECANLGENAADNPANWQIQLPIAVTDATGTLLKIVLQGDPAMSLIGVDASE

LYFMSYEERSALRRRHIFQRHRVVCKMSQYRSFAQYPLYEVVRMDLF
